# Social selection in historical time: The case of tuberculosis in South Korea after the East Asian financial crisis

**DOI:** 10.1371/journal.pone.0217055

**Published:** 2019-05-16

**Authors:** Hongjo Choi, Haejoo Chung, Carles Muntaner

**Affiliations:** 1 Department of Research and Development, Korean Institute of Tuberculosis, Korean National Tuberculosis Association, Cheongju, Republic of Korea; 2 BK21PLUS Program in Embodiment: Health-Society Interaction, Department of Public Health Sciences, Graduate School, Korea University, Seoul, Republic of Korea; 3 School of Health Policy & Management, College of Health Science, Korea University, Seoul, Republic of Korea; 4 Bloomberg Faculty of Nursing, University of Toronto, Toronto, Ontario, Canada; 5 Dalla Lana School of Public Health, University of Toronto, Toronto, Ontario, Canada; University of Maryland School of Medicine, UNITED STATES

## Abstract

The perspectives of social selection and causation have long been debated. Social selection theory is as “social” as social causation theory, since all diseases are social and no biological process occurs outside society. To identify the social selection pathway and historical juncture affected by socioeconomic and political changes, we investigated the reciprocal impact of suffering from tuberculosis (TB) on the current socioeconomic position (SEP), stratified by childhood SEP. We also examined the extent to which the social consequences of ill health changed since the East Asian economic downturn. Data were collected for 2007–2012 from the Korea National Health and Nutritional Examination Survey. To identify associations between TB history and current household income (HHI), we constructed an ordinal logistic regression model adjusted for covariates, including age, gender, educational attainment, and job status. We adopted a recursive regression model to examine trend changes in this association from 1980–2012 to 2003–2012. Of 28,136 participants, 936 had experienced TB. In the first ordinal logistic regression, the TB group was more likely to have lower HHI than the non-TB group. The odds ratios (ORs) increased from 1.30 (1980–2012) to 1.86 (2003–2012) for the TB group, increasing their probability of having low HHI. Among the low childhood SEP group, the TB group’s probability of having low HHI was 1.35 (95% confidence interval [CI]: 1.16–1.57) during 1980–2012, which increased to 2.01 (95% CI: 1.37–2.95) during 2003–2012. For the high childhood SEP group, the TB group’s OR range fluctuated, similar to that for the non-TB group. The results support the social selection pathway from TB history to adverse impact on current SEP. Our study identified downward social mobility due to TB history among the low childhood SEP group. Moreover, negative social consequences deteriorated since the East Asian economic crisis.

## Introduction

### Social selection model and debate to explain health inequality

The perspectives of social selection and causation have long been debated [1]. While there are several definitions of the social selection model, social selection generally means that the biologic traits of individuals determine their social positions, particularly for those with mental disorders or disabilities [2, 3]. The model is usually studied under the concept of social mobility, along with intergenerational and intra-generational mobility [4, 5]. Although the social selection model plays a relatively minor role in the field of health inequality [6, 7], several empirical studies have examined upward or downward social mobility based on the social selection hypothesis [8–10].

However, we believe that social selection theory is as “social” as social causation theory, since all diseases are social, and no biological process occurs outside society. Moreover, in comparison with the biologic explanation of social selection, West (1991) argued that social discrimination, including stigmatization or unhealthy status labeling, could be a mechanism to explain social selection [5]. In particular, tuberculosis (TB) was conceptualized as the cause and consequence of socioeconomic position (SEP) [11]. However, there are few empirical studies identifying social mobility for those suffering of TB in a model with temporality.

### Importance of historical time

Isaac and Griffin (1989) criticized the studies that used pooled regression or subunits of the time parameter as dummy variables could ensure a “separation of theory and history,” consequently presenting “ahistorical” results. They argued that “ahistoricism” was generated from an arbitrary pooling of the timeframe, which includes “war and peace, and depression and prosperity” [12]. Alternatively, Griffin and Isaac (1992) suggested “recursive regression,” constructed based on theoretically and historically driven methods.

### The East Asian financial crisis and tuberculosis in South Korea

Among the Organisation for Economic Co-operation and Development (OECD) countries, South Korea’s TB burden is by far the highest. One hypothesis is the “Korean War” thesis, wherein scholars argue that TB was widespread during the Korean War (1950–1953), and the disease progressed in many of the now-elderly population from latent TB infection when certain conditions were met [13, 14]. These could explain the absolute burden of TB in South Korea but not why it has recently stagnated.

To understand this, we paid limited attention to a “social” explanation. From a global perspective, TB is conceptually considered as a cause or consequence of SEP [15]. These arguments are not new in public health studies. In the second half of the 20th century, economic growth was considered an important factor in eliminating the global burden of infectious diseases [16, 17]. An economic downturn could influence disease susceptibility and treatment consequences by increasing both social inequality among the vulnerable population and deterioration of health infrastructure, which could also influence the transmission of infectious disease [18]. In previous TB research, approaches similar to that of McKeown’s thesis of “fundamental causes” were used to investigate the relationship between economic development and TB morbidity. In Latin American countries, economic growth was associated with low TB mortality; however, this association was eliminated when models were adjusted for the increase in income inequality [19]. The International Monetary Fund (IMF) promoted cuts to social spending in recipient countries, including spending for health, which had a negative effect on TB mortality in Eastern European countries [20, 21]. These studies have consistently emphasized the impact of economic development and crisis on the TB burden, even if the mechanisms involved are still unclear in these macro-level studies.

Therefore, it would be an appropriate approach to scrutinize the Korean context based on the conceptual perspective of TB, along with social selection or causation hypothesis, as well as macro-level determinants. In addition, studies on financial crises used macro-comparative designs but paid little attention to individual SEP. Because group-level correlations are not always consistent with individual-level relationships [22], it is important to identify the compositional effects in an individual-level study.

The study thus aims to investigate the impact of TB suffering on current household income, stratified by childhood SEP. The study also examines the extent to which the social consequences of ill health have changed since the East Asian economic downturn.

## Methods

### Study population

To identify previous TB patients, data were collected for 2007–2012 from the Korea National Health and Nutritional Examination Survey, which is “a nationwide cross-sectional survey conducted every year, and its target population comprises nationally representative non-institutionalized civilians in Korea” [23]. We generated a pooled dataset and a retrospective cohort model by considering the temporal order of variables from the cross-sectional surveys.

**Fig 1:**
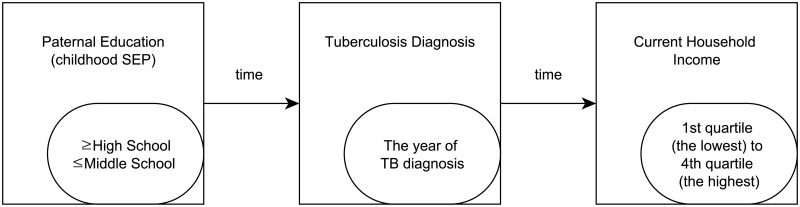
Temporal ordering of three main variables.

We measured parental educational attainment as a proxy of previous SEP, which reflects the childhood SEP before TB diagnosis, self-reported experiences of previous TB diagnoses, and current household income (HHI) as the proxy of post SEP that is considered as current SEP after TB treatment (Fig 1). All three variables were measured at the same time; however, we could logically build a temporal order from paternal education to TB diagnosis and then to current HHI. We constructed a retrospective cohort model using this temporal ordering. In addition, patients who had experienced active TB reported their age at the first diagnosis of this disease. In the dataset, the number of patients with TB gradually increased every year until 1980 and decreased thereafter. Thus, we selected 1980 as the first year of study to be close to the TB incidence pattern of the population of South Korea that gradually decreased since 1975. As a result, only people aged 32 years and above in 2012 were included in the study, because they would have been born in or after 1980.

### Measurements

Lifetime TB diagnosis is a main variable in the study. This term is applicable to an individual answering, “yes” to the following questions: (1) “Have you ever had pulmonary TB?” (2) “On examining you, has a physician ever diagnosed pulmonary TB?” (3) “How old were you when you had pulmonary TB?” Paternal education was categorized into two groups: ≤ middle school and ≥ high school. If, for any individual, data on these variables were only partially available, that individual was excluded from the study. Thus, the independent variable was constructed in combination with paternal education and TB history using the following dummy variables: level of paternal education (High and Low) and lifetime TB history (TB or non-TB). The combination of variables non-TB and Low was described as Low SEP/non-TB, TB and Low as Low SEP/TB, TB and High as High SEP/TB, and non-TB and High as High SEP/non-TB. As the outcome variable, the current HHI was transformed into quartile HHI, with the fourth quartile being the highest income group. Other covariates included age, gender, educational attainment, and job status, which were categorized as follows: age into four groups (32–39, 40–49, 50–59, and 60+ years), gender into two groups (female and male), educational attainment into three groups (≤ middle school, high school, and ≥ university), and job status into six groups (owner/self-employed, permanent, temporary, part-time, unemployed/economically inactive/household worker, and unknown).

### Data analysis

The lifetime TB history for all variables was estimated and the statistical significance of the association was tested with Pearson’s chi-squared test. To identify associations between TB history and the current HHI, we constructed an ordinal logistic regression model, and HHI was reverse coded (0: highest HHI; 3: lowest HHI). This model was adjusted for covariates, namely age, gender, own educational attainment, and job status. Because the youngest age at TB diagnosis was 27 years old among participants, the effect of educational progress after TB treatment was minimized. Next, we constructed the recursive analysis model. In the first analysis, we used data on individuals diagnosed with TB between 1980 and 2012 and on all individuals without TB (i.e., non-TB). We excluded individuals with TB diagnosed in 1980 from the second analysis; in each subsequent analysis, we excluded individuals diagnosed with TB in each subsequent year after 1980. The final analysis used data on all individuals diagnosed with TB between 2003 and 2012 and on all individuals without TB. Consequently, a set of odds ratios (ORs) was serially calculated for each timeframe from 1980–2012 to 2003–2012. To identify the general trend, we combined the childhood SEP group at the first analysis and, subsequently, childhood SEP-stratified analysis was conducted. The serial ORs were plotted in a graph to identify any structural breaks in the associations. To understand the downward social mobility, the model was also applied within the same strata of childhood SEP. All data analyses were performed using STATA/MP version 13.0 (StataCorp, College Station, TX, USA) to account for sampling weights.

## Results

**Fig 2:**
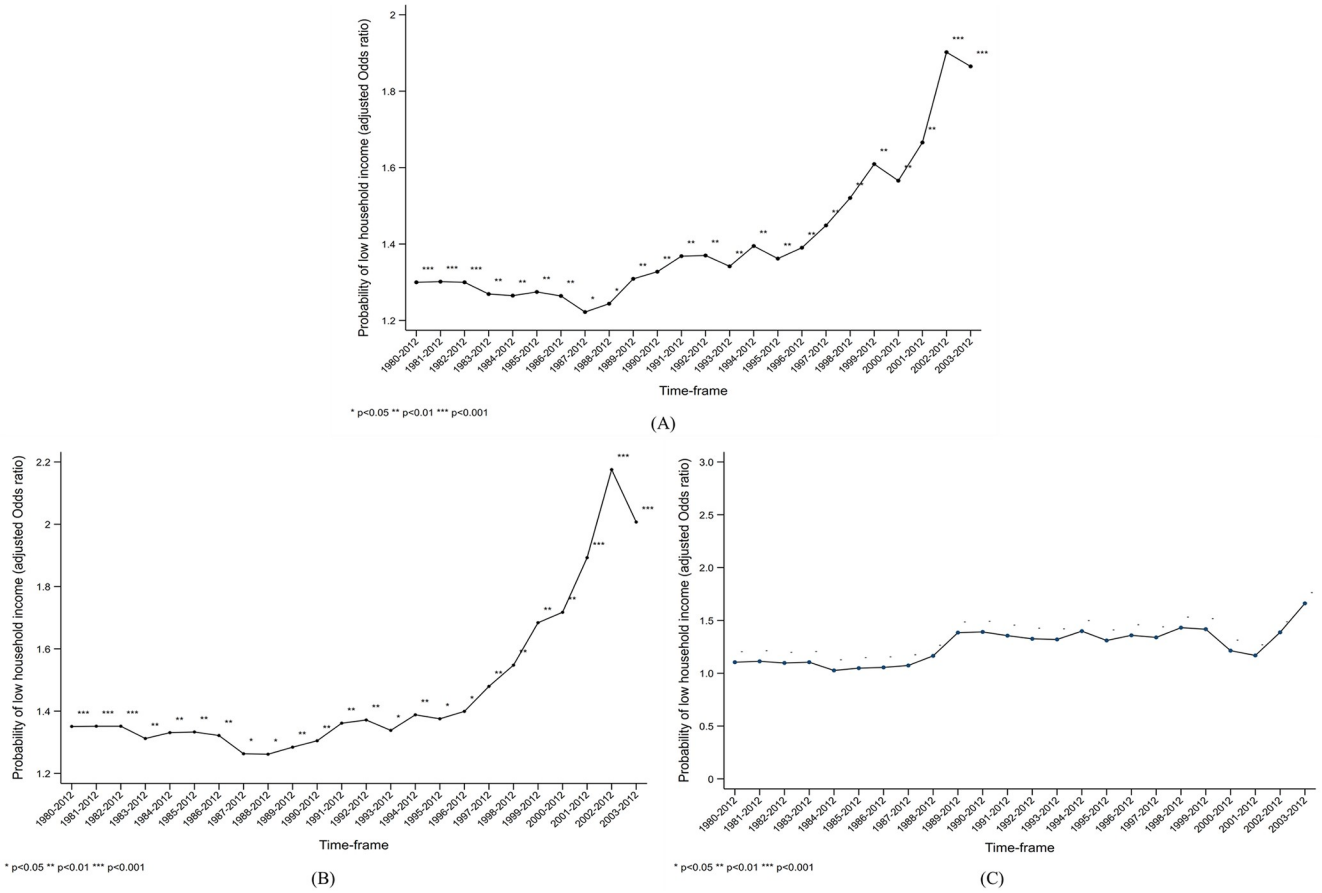
Probability of low household income between tuberculosis and non-tuberculosis group and its historical trend. (A) All study population included. (B) Comparison within low childhood socioeconomic position strata (C) Comparison within high childhood socioeconomic position strata.

There were 28,136 participants, and 936 (3.3%) among them had experienced TB in their lifetime. The general characteristics of all variables are listed in Table 1. The male and older age groups experienced TB more frequently in their lifetime, and the differences with their counterparts are statistically significant. The proportion of participants with TB is significantly higher in the lowest quartile of HHI than in the highest. Other variables, including paternal education, job status, and education, are not differentiated by lifetime TB history. In the first ordinal logistic regression, the TB group was more likely to have lower HHI than the non-TB group. After eliminating data on individuals diagnosed with TB between 1980 and 2002 (timeframe: 2003–2012), the same trend was observed after adjusting for age, gender, educational attainment, and job status. The ORs increased from 1.30 (1980–2012) to 1.86 (2003–2012) for the TB group, thus increasing their probability of having low HHI (Table 2). Moreover, among the low childhood SEP group, the probability of the TB group having low HHI was 1.35 (95% confidence interval [CI]: 1.16–1.57) in the 1980–2012 timeframe), but increased to 2.01 (95% CI: 1.37–2.95) in the 2003–2012 timeframe (Table 3). The analysis of the high childhood SEP group showed that the probability of the TB group having low HHI increased slightly from 1.10 (95% CI: 0.81–1.51) and 1.66 (95% CI: 0.85–3.27) for the 1980–2012 and 2003–2012 timeframes, respectively, but no difference exists between the TB and non-TB groups (Table 4). All point estimates were combined in a plot graph to present historical trends. First, we identified that TB history increased the probability of having low HHI, but the range of ORs was limited between 1.22 and 1.39 before 1997–2012. However, after excluding the data for 1996, the OR began to increase, reaching 1.90 in the 2002–2012 timeframe (Fig 2A). Therefore, the structural break point from the analysis was indicated in 1997. Moreover, a similar trend was observed within the low childhood SEP group (Fig 2B). However, within the high childhood SEP group, the range of ORs fluctuated for the TB group, which was not significantly different from that of the non-TB group (Fig 2C).

**Table 1:** Baseline characteristics of all variables by lifetime tuberculosis histories (n = 28,136).

Variables		TB diagnosis in lifetime				p-value
		No	%	Yes	%	
Gender	Male	11,271	95.6	525	4.5	<0.001
	Female	15,929	97.5	411	2.5	
Age	32–39	4,673	97.3	132	2.8	0.007
	40–49	6,146	96.3	236	3.7	
	50–59	5,773	96.3	224	3.7	
	60–	10,618	96.9	344	3.1	
Paternal education	High school or above	5,600	96.8	186	3.2	0.594
	Middle school or below	21,600	96.6	750	3.4	
Own Education	Middle school or below	11,661	96.8	392	3.3	0.745
	High school	8,425	96.7	289	3.3	
	University or above	7,114	96.5	255	3.5	
Job status	Owner/self-employed	5,841	96.7	201	3.3	0.944
	Permanent	6,355	96.5	228	3.5	
	Temporary	1,294	96.5	47	3.5	
	Daily	1,156	96.7	40	3.3	
	Unemployed/economic inactive/house worker	12,538	96.8	419	3.2	
	Unknown	16	94.1	1	5.9	
Current Household income	1st (the lowest)	5,851	96.3	224	3.7	0.013
	2nd	6,873	96.4	256	3.6	
	3rd	7,218	96.7	248	3.3	
	4th (the highest)	7,258	97.2	208	2.8	

**Table 2:** The odds ratios of low household income among tuberculosis and non-tuberculosis group by periods.

Time-frame	aOR*	95% CI**	p-value
1980–2012	1.30	1.13–1.49	<0.001
1981–2012	1.30	1.13–1.50	<0.001
1982–2012	1.30	1.13–1.50	<0.001
1983–2012	1.27	1.09–1.47	0.002
1984–2012	1.26	1.09–1.47	0.003
1985–2012	1.27	1.09–1.49	0.002
1986–2012	1.26	1.08–1.48	0.004
1987–2012	1.22	1.04–1.44	0.017
1988–2012	1.24	1.05–1.47	0.011
1989–2012	1.31	1.10–1.55	0.002
1990–2012	1.33	1.11–1.59	0.002
1991–2012	1.37	1.13–1.65	0.001
1992–2012	1.37	1.13–1.66	0.001
1993–2012	1.34	1.09–1.65	0.005
1994–2012	1.39	1.13–1.72	0.002
1995–2012	1.36	1.09–1.70	0.006
1996–2012	1.39	1.10–1.76	0.006
1997–2012	1.45	1.14–1.85	0.003
1998–2012	1.52	1.18–1.96	0.001
1999–2012	1.61	1.23–2.11	0.001
2000–2012	1.57	1.18–2.07	0.002
2001–2012	1.67	1.24–2.24	0.001
2002–2012	1.90	1.39–2.60	<0.001
2003–2012	1.86	1.34–2.60	<0.001

* aOR: adjusted odds ratio, adjusted for age, gender, own education and job status

** CI: confidence interval

**Table 3:** The odds ratios of low household income among tuberculosis and non-tuberculosis group by periods within low childhood socioeconomic position strata.

Time-frame	aOR*	95% CI**	p-value
1980–2012	1.35	1.16–1.57	<0.001
1981–2012	1.35	1.16–1.58	<0.001
1982–2012	1.35	1.15–1.58	<0.001
1983–2012	1.31	1.11–1.55	0.001
1984–2012	1.33	1.12–1.58	0.001
1985–2012	1.33	1.12–1.58	0.001
1986–2012	1.32	1.11–1.57	0.002
1987–2012	1.26	1.05–1.51	0.011
1988–2012	1.26	1.05–1.52	0.014
1989–2012	1.28	1.06–1.55	0.010
1990–2012	1.30	1.07–1.59	0.009
1991–2012	1.36	1.11–1.67	0.003
1992–2012	1.37	1.11–1.70	0.004
1993–2012	1.34	1.06–1.69	0.013
1994–2012	1.39	1.09–1.76	0.007
1995–2012	1.38	1.07–1.77	0.014
1996–2012	1.40	1.07–1.83	0.015
1997–2012	1.48	1.12–1.95	0.006
1998–2012	1.55	1.16–2.07	0.003
1999–2012	1.68	1.23–2.31	0.001
2000–2012	1.72	1.24–2.38	0.001
2001–2012	1.89	1.36–2.64	<0.001
2002–2012	2.18	1.52–3.10	<0.001
2003–2012	2.01	1.37–2.95	<0.001

* aOR: adjusted odds ratio, adjusted for age, gender, own education and job status

** CI: confidence interval

**Table 4:** The odds ratios of low household income among tuberculosis and non-tuberculosis group by periods within high childhood socioeconomic position strata.

Time-frame	aOR*	95% CI**	p-value
1980–2012	1.10	0.81–1.51	0.534
1981–2012	1.11	0.80–1.54	0.523
1982–2012	1.10	0.78–1.54	0.593
1983–2012	1.10	0.78–1.56	0.571
1984–2012	1.03	0.72–1.47	0.888
1985–2012	1.05	0.72–1.52	0.805
1986–2012	1.06	0.72–1.54	0.781
1987–2012	1.07	0.73–1.58	0.718
1988–2012	1.16	0.79–1.71	0.438
1989–2012	1.38	0.93–2.06	0.111
1990–2012	1.39	0.93–2.09	0.111
1991–2012	1.36	0.87–2.11	0.178
1992–2012	1.33	0.86–2.05	0.203
1993–2012	1.32	0.84–2.07	0.229
1994–2012	1.40	0.89–2.20	0.149
1995–2012	1.31	0.82–2.09	0.256
1996–2012	1.36	0.84–2.20	0.212
1997–2012	1.34	0.80–2.24	0.265
1998–2012	1.43	0.84–2.43	0.185
1999–2012	1.42	0.80–2.50	0.230
2000–2012	1.21	0.68–2.16	0.511
2001–2012	1.17	0.61–2.24	0.640
2002–2012	1.39	0.72–2.68	0.329
2003–2012	1.66	0.85–3.27	0.141

* aOR: adjusted odds ratio, adjusted for age, gender, own education and job status

** CI: confidence interval.

## Discussion

To the best of our knowledge, this is the first study to elucidate the social selection pathway of TB and SEP, as well as its interrelation with the economic crisis. We hypothesized that downward social mobility might be observed among the group with TB history. The study results partially explain mobility, because TB history was associated with low HHI within the low childhood SEP group but not the high childhood SEP group. Among those with low childhood SEP, it was more difficult for those with TB than without TB to have a high HHI, and this became even more difficult after the East Asian economic crisis of 1997.

The results are consistent with those of other studies focusing on the relationship between SEP and health status. This relationship was previously evaluated based on the social causation and social selection pathways [24–26]. Dohrenwend et al. [24] focused on mental disorders and identified the effect of SEP on health status, as well as the reverse pathway. Mulatu and Schooler [25] studied the association between self-reported health scales and SEP measures. More recently, several empirical studies investigated reciprocal pathway and focused on unstable working conditions as a consequence of ill health [27–29]. TB researchers have also focused on the impact of TB history on social inequality but found little empirical evidence [15, 30].

Nevertheless, there is considerable evidence supporting our results. First, the direct and indirect cost from the time lost by a patient seeking and receiving care might be a driver of social consequences. A meta-analysis conducted in low- and middle-income countries showed that the indirect cost arising mainly from income loss due to TB treatment was approximately 60% of the total financial burden of receiving TB care [31]. The other study also identified a large amount of productivity losses due to TB care in high income countries [32]. Within the South Korean health insurance system, co-payment and out-of-pocket medical cost comprised more than 50% of total cost [33], and catastrophic health expenditure was also a significant issue [34]. The duration of TB treatment is at least six months, but the period can extend to more than two years for patients with drug-resistant TB. Therefore, a relatively long treatment period and weak health insurance coverage could increase a patient’s costs.

Second, the precarious labor market and low social protection would cause economic suffering for TB patients during the treatment period and immediately after treatment completion. During the treatment period, various employment challenges could emerge, such as the use of sick leave, job and income loss, and the threat of being fired. In particular, studies reported the existence of a vulnerable Korean labor market with high level of precariousness and its association with health status [35, 36]. With insufficient social protection, the adverse effects of the financial burden would be extended. After the Korean economic crisis, the proportion of unemployed persons, number of homeless persons [37], and income inequality [38] suddenly increased. GDP per capita in Korea decreased at -5.9% in the first quarter of 1998 and rapidly recovered in the fourth quarter of 1998 [39]. The unemployment rate in Korea was around 2% in 1997, but increased to 8.7% in 1999 and slightly stabilized in 2000, but never returned to the level before the economic crisis [37, 40]. Even though some indicators are relatively stable, income inequality in Korea has been increasing since the economic crisis, and the Gini index is still high [41]. Several previous studies highlight the adverse effect of low SEP on health after economic crises [39, 42–48]. In our study, the reciprocal effect of TB history on SEP exacerbated after the financial crisis. This result implies that, after the financial crisis, it has become more difficult for TB sufferers to recover their income level after the completion of TB treatment because of the precarious labor market situation. On the other hand, there were no differences in current HHI between the TB and non-TB groups if childhood SEP was high. This suggests that people with relatively high SEP are less likely to suffer from economic strain during TB treatment and after treatment completion. This is because the previous high SEP may buffer the socioeconomic burden derived from the disease.

Third, TB might cause long-term TB sequelae, including impaired pulmonary function and respiratory symptoms [49]. In addition, social stigma and discrimination would play an important role in hindering economic recovery after TB treatment, which persisted for long periods among those with TB history [50].

In our study, a recursive analysis was performed with a moving set of historical periods rather than a fixed set. Several TB studies have been conducted without considering historical time as a contextual dynamic [51, 52]. A study to identify the birth cohort effect of latent TB infection used five-year interval timeframes without concern about the rationality to fix a specific period [52]. Lifetime TB prevalence was also used as an outcome variable in a study conducted in South Africa [51]; however, the lifetime might not only be a dynamic period but also include politically unstable periods such as the apartheid and democratization in the South African context. Because a social phenomenon is closely linked with historical time, causal pathways in pooled analyses should be interpreted with caution [53]. From our results, the pooled analysis presented well the reciprocal pathway from TB history to low SEP, but nothing more. Understanding and considering historical time in our recursive analysis would be useful to identify a break or turning point of downward social mobility, such as the economic crisis in our case.

This study has several limitations. Firstly, childhood SEP was measured as paternal educational attainment, which was not the same as post SEP (own HHI): the best way to do this is to compare previous HHI before TB diagnosis with post HHI after TB treatment. However, our dataset was insufficient to perform a more robust comparison. Therefore, we considered paternal educational attainment as a proxy for childhood SEP, because paternal SEP is well correlated with post SEP, regardless of the TB history in our dataset. Second, the study may have a selection bias. Many patients with TB had died before the survey period and, consequently, these cases could be included in the survey. From the dataset, TB cases gradually increased since the 1930s and decreased after 1980. If we consider the real trend of TB incidence in South Korea, many TB cases diagnosed during 1930–1979 were not captured in the survey [54]. To reduce this bias, we chose 1980 as the first year of the study. Third, we controlled for job status as a potential confounder, which may be an underestimation of the social selection. However, the same job status between study participants could not place them in the same position, because age distribution was wide and individuals who started to work after the economic crisis would be in a more vulnerable working position. Therefore, we considered it would be a potential confounder in the association between TB history and household income.

The results from this study support the social selection pathway from TB history to the adverse impact on current SEP. Our study also identified the existence of downward social mobility due to TB history among the low childhood SEP group. Moreover, the negative social consequence has exacerbated since the East Asian economic crisis. Consequently, the study provides evidence to support the timely implementation of the “End TB Strategy,” which includes elimination of catastrophic cost as a target indicator in conjunction with the implementation of poverty alleviation strategies and social protection measures to eliminate TB [30]. However, our results should be interpreted with caution because of the time lapse between TB history and HHI. Therefore, low HHI might be a cause of TB among some study participants who still presented low HHI at the end point, because we could not measure the previous HHI from our dataset. As such, further studies are still required for a deeper understanding of the causal relationship from TB history to low SEP based on the social selection pathway. Both qualitative and quantitative studies would be required to identify the causal pathways underlying this relation. In particular, these studies would provide a good way to identify which socioeconomic barriers, such as job insecurity and indirect care cost, cause TB patients to become poor during or after the TB treatment period and how these barriers function. Finally, policy interventions should focus on reducing the harmful effects of economic crises on health concerns and adapting countercyclical policies in the labor market and social services.
